# Accelerating Prediction of Malignant Cerebral Edema after Ischemic Stroke with Automated Image Analysis and Explainable Neural Networks

**DOI:** 10.1007/s12028-021-01325-x

**Published:** 2021-08-20

**Authors:** Hossein Mohammadian Foroushani, Ali Hamzehloo, Atul Kumar, Yasheng Chen, Laura Heitsch, Agnieszka Slowik, Daniel Strbian, Jin-Moo Lee, Daniel S Marcus, Rajat Dhar

**Affiliations:** 1) Department of Electrical and Systems Engineering, Washington University in St. Louis;; 2) Department of Neurology, Washington University School of Medicine;; 3) Department of Emergency Medicine, Washington University School of Medicine;; 4) Department of Neurology, Jagiellonian University Medical College, Krakow, Poland;; 5) Department of Neurology, Helsinki University Hospital, Helsinki, Finland;; 6) Department of Radiology, Washington University School of Medicine

**Keywords:** brain edema, cerebral infarction, brain CT scan, deep learning, early diagnosis

## Abstract

**Background/Objective:**

Malignant cerebral edema is a devastating complication of stroke, resulting in deterioration and death if hemicraniectomy is not performed prior to herniation. Current approaches for predicting this relatively rare complication often require advanced imaging and still suffer from suboptimal performance. We performed a pilot study to evaluate whether neural networks incorporating data extracted from routine CT imaging could enhance prediction of edema in a large diverse stroke cohort.

**Methods:**

An automated imaging pipeline retrospectively extracted volumetric data, including CSF volumes and hemispheric CSF volume ratio, from baseline and 24-hour CTs performed in participants of an international stroke cohort study. Fully connected and long short-term memory (LSTM) neural networks were trained using serial clinical and imaging data to predict those who would require hemicraniectomy or die with midline shift. The performance of these models were tested, in comparison with regression models and the EDEMA score, using cross-validation to construct precision-recall curves.

**Results:**

Twenty of 598 patients developed malignant edema (12 required surgery, 8 died). The regression model provided 95% recall but only 32% precision (area under precision-recall curve [AUPRC] 0.74), similar to the EDEMA score (precision 28%, AUPRC 0.66). The fully connected network did not perform better (precision 33%, AUPRC 0.71) but the LSTM model provided 100% recall, 87% precision (AUPRC of 0.97) in the overall cohort and the subgroup with NIHSS ≥ 8 (p=0.0001 vs. regression and fully connected models). Features providing the most predictive importance were the hemispheric CSF ratio and NIHSS score measured at 24-hours.

**Conclusion:**

A LSTM neural network incorporating volumetric data extracted from routine CTs identified all cases of malignant cerebral edema by 24-hours after stroke, with significantly fewer false positives than a fully connected neural network, regression model and the validated EDEMA score. This preliminary work requires prospective validation but provides proof-of-principle that a deep learning framework could assist in selecting patients for surgery, prior to deterioration.

## INTRODUCTION

Cerebral edema develops in the hours to days after acute ischemic stroke and may result in midline shift and cerebral herniation. Even though a leading cause of death and deterioration, only a small proportion of all stroke patients will develop this life-threatening complication. As deterioration is usually delayed by a few days after stroke, an important opportunity for early detection and intervention exists [1]. Decompressive hemicraniectomy (DHC), if performed prior to deterioration and within 48 hours, dramatically reduces mortality and improves chances of functional recovery [2]. Accurately predicting which hemispheric stroke patients will go on to develop malignant edema is therefore of vital importance in acute stroke care [3].

However, the current approach to edema detection is primarily reactive, waiting for signs of mass effect and deterioration to manifest before intervening. This paradigm is driven by the poor predictive value of clinical variables; for example, stroke severity (measured by NIHSS) is generally higher in those who will develop malignant edema, but no specific threshold adequately distinguishes groups or permits confident triage to surgery [4]. Brain imaging provides the opportunity to visualize the early effects of edema before decompensation. Signs of early infarct-related hypodensity, such as lower ASPECT score, increase the likelihood of edema but cannot reliably inform decision-making in isolation [5]. While larger infarct lesion volume is a risk factor for greater edema, there is significant variability in midline shift and risk of deterioration between patients with equivalent size strokes [6, 7]. Therefore, several approaches at quantifying early edema-related injury have been investigated [8, 9].

The volume of CSF relative to total cranial volume, termed *intracranial reserve*, is emerging as a useful biomarker, with lower reserve on baseline imaging increasing the risk for subsequent edema-related decompensation [10, 11]. Assessment of findings on repeat CT within 24-hours may allow refined prognostication as edema develops but prior to deterioration. However, most existing measures are either qualitative (e.g. effacement of basal cisterns) or require manual measurement (e.g. infarct volume, midline shift). We previously developed a nomogram, termed the EDEMA score, which incorporates manually extracted imaging features to assist in edema prediction [12]. We have developed imaging algorithms to extract meaningful quantitative measures of edema from routine CTs, based around displacement of CSF volume [13, 14]. We demonstrated that the proportion of total CSF displaced by 24-hours provides a reliable biomarker of edema severity [15]. By incorporating CSF volumetrics from baseline and 24-hour CTs into regression-based models, we were able to improve the detection of which stroke patients would develop malignant edema [16]. However, the precision of this prediction remained relatively low, that is for every true case of malignant edema identified two false positives would also be mislabeled.

We now propose a pilot study evaluating the impact of two significant innovations to this imaging-based prediction algorithm: the first is to incorporate hemispheric CSF ratio (i.e. the ratio of CSF volumes between the two hemispheres), not just total CSF displacement, as a targeted feature that we have recently developed and that appears more specific to stroke-related edema [17]; the second is to employ neural networks that can integrate serial clinical and imaging features in complex, dynamic, and non-linear ways to enhance prediction beyond what is capable by traditional regression models. In this study, we employ these two innovations to develop and internally validate an automated deep learning-based imaging and prediction framework to enhance the prediction of malignant edema after stroke.

## METHODS

### Study Participants and Clinical Data

Patients enrolled in an international prospective in-patient stroke cohort (the Genetics of Neurological Instability after Ischemic Stroke, *GENISIS*) study between 2008 and 2017 were retrospectively evaluated for eligibility. All participants presented within six hours of stroke onset. We selected those with baseline CT within 12 hours of onset and a follow-up CT within 48 hours from three sites. At two sites, it was standard protocol to obtain a repeat CT at 24-hours after thrombolytic and/or endovascular therapies. At the third, it was performed at the stroke physician’s discretion and for any deterioration or concerns for neurological complications. We excluded participants if onset time was unknown, if baseline CT already showed well-developed infarction (suggesting that time of stroke onset was likely earlier than annotated; other acute stroke-related hypodensity was acceptable), if the stroke was located in the brainstem or cerebellum, or if the final discharge diagnosis was not stroke.

NIHSS scores were obtained at baseline (within six hours of last known well) and at 24-hours. Serum glucose and blood pressure were obtained on presentation. All participants provided informed consent. They were followed prospectively for neurological deterioration or death during hospital admission. Surgery (DHC) was considered in cases of clinical deterioration with midline shift and not pre-emptively prior to deterioration. Our primary endpoint was the development of malignant edema leading to either DHC and/or death in the presence of midline shift of 5-mm or greater [12]. This retrospective imaging sub-study was approved by the coordinating site’s institutional review board.

### Imaging Analysis

Both the baseline CT on admission and follow-up CT performed closest to 24-hours (but before DHC), were processed using an image analysis workflow to extract quantitative metrics, as described previously [18]. Intracranial and CSF volumes were obtained using an automated algorithm and the intracranial reserve was calculated as the proportion of intracranial volume comprised by CSF (i.e. CSF volume divided by cranial volume). ΔCSF was calculated as the percent change in CSF volume from baseline to follow-up CT [14]. The midline was delineated on all axial CT slices using a registration approach that aligned each CT to a brain atlas [19].

**Figure 1:**
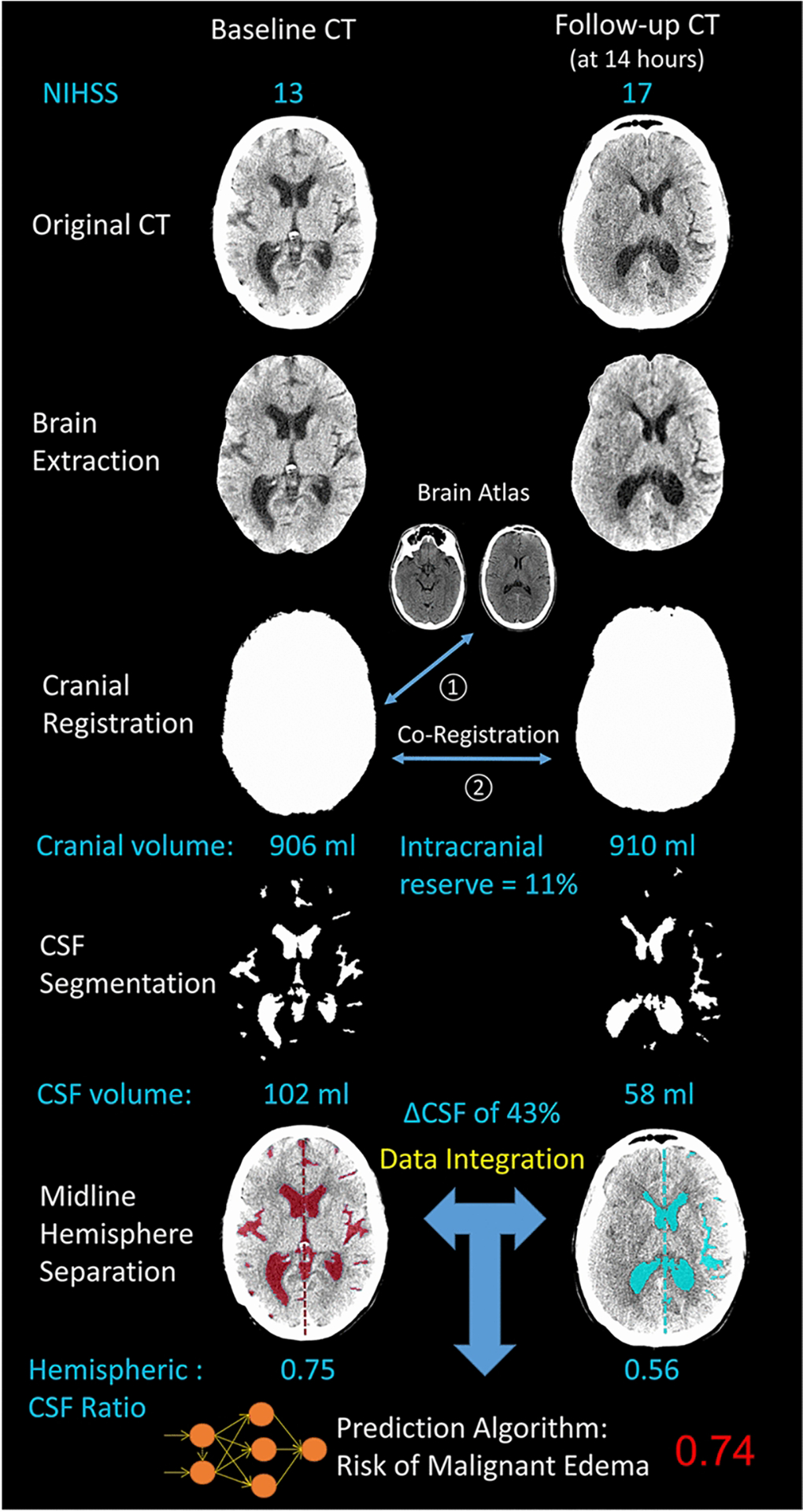
Automated workflow used to extract CSF volumetrics from baseline and follow-up CT scans and predict risk of malignant edema: illustrated is case of a 28-year old female with diabetes who presented with baseline NIHSS of 13 and glucose of 421 mg/dl. Baseline CT within one hour of symptom onset and follow-up CT at 14-hours were analyzed. Cranial and CSF volumes were extracted and midline separation was used to calculate the hemispheric CSF ratio. The deep learning model provided a prediction of malignant edema of 0.74. She subsequently developed 7-mm of midline shift, drowsiness, and received hyperosmolar therapy followed by hemicraniectomy at 32-hours after stroke onset.

Segmented CSF was divided into hemispheric volumes using the midlines from each registered slice. The hemispheric CSF ratio was calculated as the CSF volume in the stroke-affected divided by the volume in the contralateral hemisphere; on baseline CT or when stroke lesion was not visible, the affected hemisphere was determined by the hemisphere with less CSF. This biomarker has recently been validated as a sensitive measure of edema evolution and severity [17]. Although each step in this pipeline has been tested previously, this was the first time extracting all volumetric phenotypes (ΔCSF, CSF ratio, intracranial reserve) in a single automated workflow (Figure 1).

All these imaging variables were employed as predictive features, along with clinical variables associated with edema from prior studies [20]. A single experienced investigator manually measured midline shift (at the septum pellucidum) and infarct volume (i.e. lesion-related hypodensity, visible on CT). However, we built our primary predictive models without these two manual metrics, in order to focus on imaging features that could be extracted automatically. We then compared these models to those incorporating these two important measures of edema to determine whether a fully automated approach would provide equivalent predictive performance.

### Model Development and Testing

Details on feature imputation, standardization, and means of training the prediction models are provided in the Supplemental Methods. Ten-fold nested and stratified cross validation was employed to test each model on patients not used for model training and internal validation. We evaluated the performance of four regression models that incorporated progressively more data: (1) only baseline clinical and imaging variables; (2) adding 24-hour NIHSS and ΔCSF; (3) all automated imaging variables, including hemispheric CSF ratio; (4) all variables including midline shift and infarct volume. We then compared these models to predictions from two neural networks, including a fully connected neural network and a recurrent neural network that employed a Long Short-Term Memory (LSTM) architecture. LSTM neural networks are specialized to optimize predictions with longitudinal interdependent data. Unlike conventional networks that only feed-forward information from one layer to the next, recurrent networks use loops to capture dependencies between data at sequential time points. The LSTM employs memory units to solve the vanishing gradient problem experienced by other recurrent networks [21]. They have been shown to improve predictions with longitudinal data and are gaining significant traction in predicting rare events in the critical care, neurology and stroke spheres [22–27]. Two LSTM models were constructed; our primary model incorporated only automated imaging features (i.e. cranial and CSF volume plus hemispheric CSF ratio). This was compared with a second LSTM model that also incorporated midline shift and infarct volume. Further details on training and optimizing these networks, including selection of hyper-parameters and network architecture, are provided in the Supplemental Methods.

### Statistical Analysis

The preferred metrics to evaluate prediction in imbalanced datasets where the outcome of interest is relatively rare are recall (sensitivity to detect cases) and precision, indicating what proportion of those predicted to have malignant edema actually turn out to develop it. The overall model performance was captured by area under the receiver-operating-characteristic (AUROC) and precision-recall curves (AUPRC). We selected AUPRC as our primary summary metric of performance as AUROC is overly optimistic when evaluating imbalanced datasets [28]. However, we provide all metrics of model performance, including accuracy, specificity and Brier scores, in a figure (comparing the main models) and a supplemental table (comparing all models). Bootstrapping was used to calculate the empirical p-value for the null hypothesis that there was no difference in AUPRC between the regression and neural network models. We also compared performance of the LSTM model with only automated imaging features to the LSTM incorporating midline shift and infarct volume. We also performed a sensitivity analysis in the subset of patients with NIHSS ≥ 8 to evaluate how these various machine-learning models predicted malignant edema in those with more severe strokes.

### Interpretability

In addition, we employed SHAP (SHapley Additive exPlanation) values and plots to provide enhanced global (across all subjects) and local (for each individual case) interpretability to the predictions provided by our deep learning model [29]. To provide comparison of our model to a validated edema prediction nomogram, we computed the ordinal EDEMA score for each subject, based on qualitative assessment of repeat CT imaging [12]. We also calculated the modified EDEMA score, which incorporates NIHSS [30].

## RESULTS

### Study Cohort

Out of 1799 stroke patients enrolled in the study at three sites, 759 had no imaging available and 336 had either no follow-up CT or it was performed beyond 48 hours after stroke. After exclusions, we had 616 participants with acute hemispheric strokes and paired baseline and follow-up CTs close to 24-hours. All 1238 images were processed using our automated pipeline to extract global and hemispheric CSF volumes. Eighteen were excluded for image quality or processing problems, leaving 598 subjects with quantitative data for analysis (see Supplemental Figure 2 for full patient flow). Those excluded had milder strokes (median NIHSS 5) and only two of those patients developed malignant edema. Of those included, baseline NIHSS was eight or greater in 328 (55%). Baseline CT was performed at a median of one and a half hours after stroke onset (IQR 1–3) and follow-up CT at 25 hours after onset (IQR 20–28).

### Factors associated with Malignant Cerebral Edema

**Figure 2:**
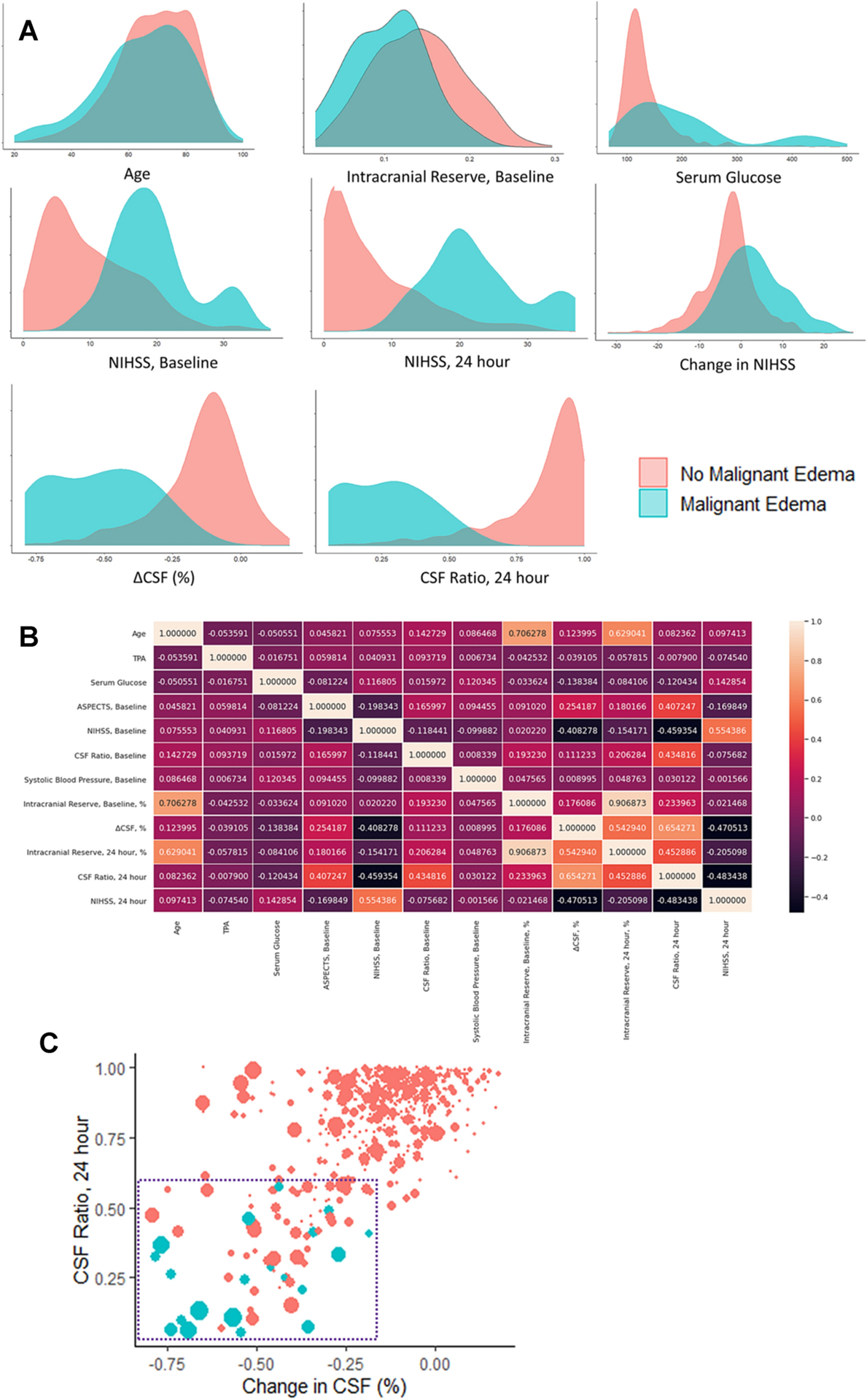
Distribution of clinical and imaging features used in the prediction models. (A) Density plots highlighting the overlap between malignant edema cases (blue) and controls (red). (B) Correlations between variables used in prediction models. (C) Scatterplot showing correlation of ΔCSF (x-axis) and the hemispheric CSF ratio on CT at 24-hours (y-axis), with size of dots representing the NIHSS score at 24-hours. The blue dashed square highlights the quadrant (ΔCSF below −20% and CSF ratio below 0.60) where all cases are found. However, many stroke patients who never develop malignant edema are also found in this region, emphasizing the difficulty of linear combinations of even highly predictive variables to discriminate rare outcomes with precision.

Twenty participants (3%) developed malignant cerebral edema requiring DHC (12) or resulting in death with midline shift of at least 5-mm (8). Median time to DHC was 2.7 days (IQR 1.7–5) and to death was 5 days (IQR 4–6). Those with malignant edema had higher NIHSS and glucose levels (Table 1). They also had a worsening of NIHSS by 24-hours while controls generally improved (mean change +3 vs. −3, p=0.0009). Although all but three of those destined for malignant edema had measurable midline shift by 24-hours, fifty others who neither died nor required surgery also had midline shift. Similarly, the median lesion volume visible at 24-hours was larger in the malignant edema group, but three had no visible hypodensity and, again, fifty controls had infarct volumes greater than 100-ml at 24-hours. ΔCSF was greater and the hemispheric CSF ratio lower at 24-hours. However, there was still significant overlap between cases and controls in each of these individual features (FIGURE 2A). There were also moderate correlations between several clinical and imaging features, including CSF ratio and both ΔCSF (r=0.65) and 24-hour NIHSS (r=−0.48, FIGURE 2B). Even linear combinations of these powerful predictive features could not adequately discriminate cases of malignant edema from many controls, as highlighted by the overlap between cases and controls in FIGURE 2C.

### Model Performance

**Figure 3:**
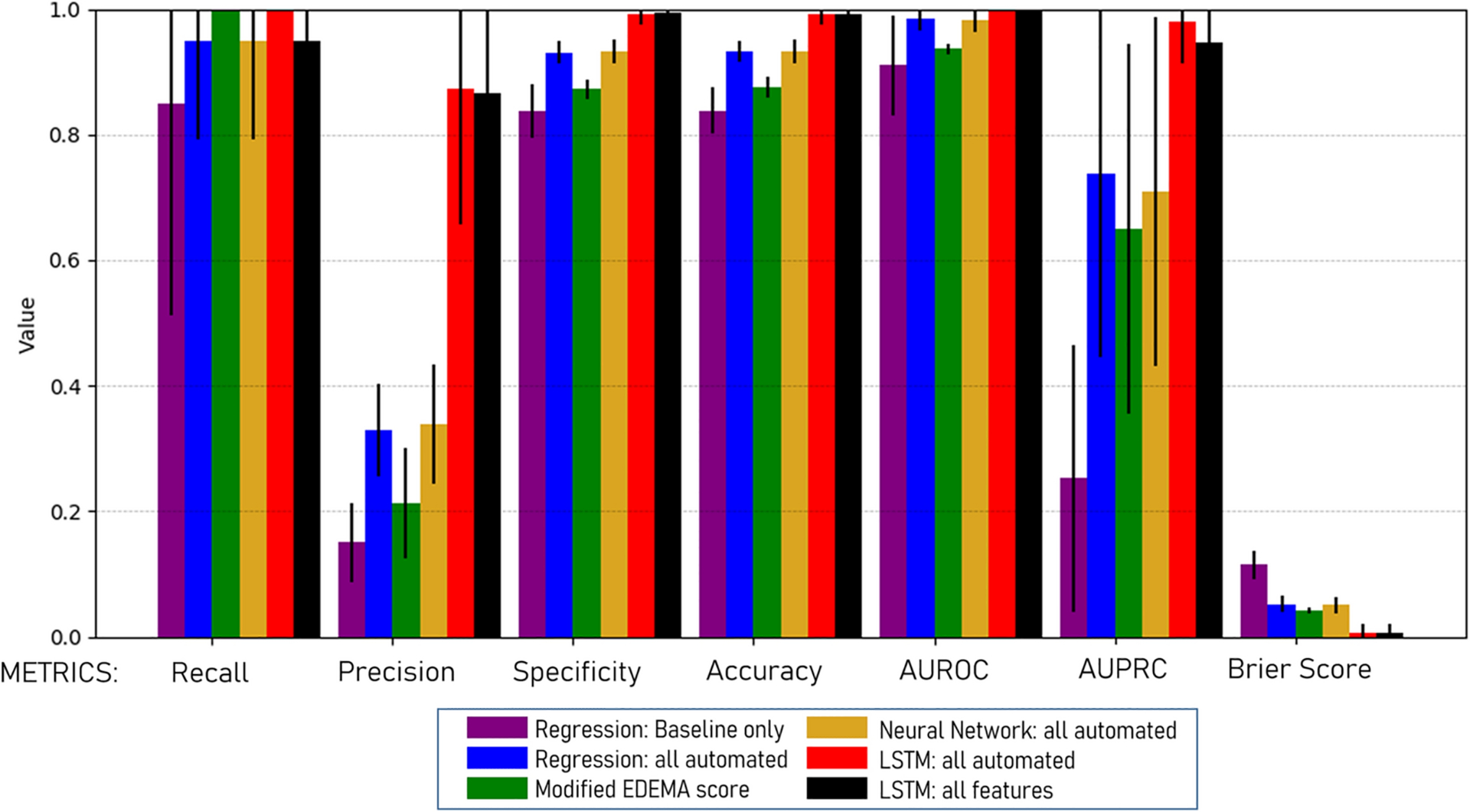
Metrics of performance (with standard deviation bars) for the main regression and neural network models, as well as the modified EDEMA score, for predicting malignant edema in all stroke patients, assessed using cross-validation.

The following variables were entered into the predictive models: age, NIHSS at baseline and 24-hours, baseline intracranial reserve, ASPECTS, glucose, systolic blood pressure, tPA treatment, CSF ratio at baseline and 24-hours and ΔCSF from baseline to 24-hours. A regression model with only eight baseline variables was able to identify 85% of cases of malignant edema (i.e. recall/sensitivity of 0.85) but with only 15% precision, meaning that almost six false positives would be identified for each case correctly labeled (AUROC 0.91, AUPRC 0.29; FIGURE 3). Incorporating 24-hour NIHSS and ΔCSF improved prediction with AUPRC increasing to 0.66 but precision remained low at 24% (see Supplemental Table 1 for full summary of model results). Adding hemispheric CSF ratio increased recall to 95% but precision remained only 32% (AUROC 0.98, AUPRC 0.74). That is, two false positives would still be designated for each correct case of malignant edema identified by this regression model. Adding midline shift and infarct volume to the regression model did not improve sensitivity and had persistently low precision (34%, p=0.00002 for equivalence to regression model with only automated data). In comparison, the EDEMA score (at a threshold of 4) provided similar sensitivity (90%) with low precision (28%) to predict malignant edema (AUPRC of 0.66). The modified EDEMA score was able to obtain 100% sensitivity (at a threshold of 5) but with only 22% precision (AUPRC of 0.68).

**Figure 4:**
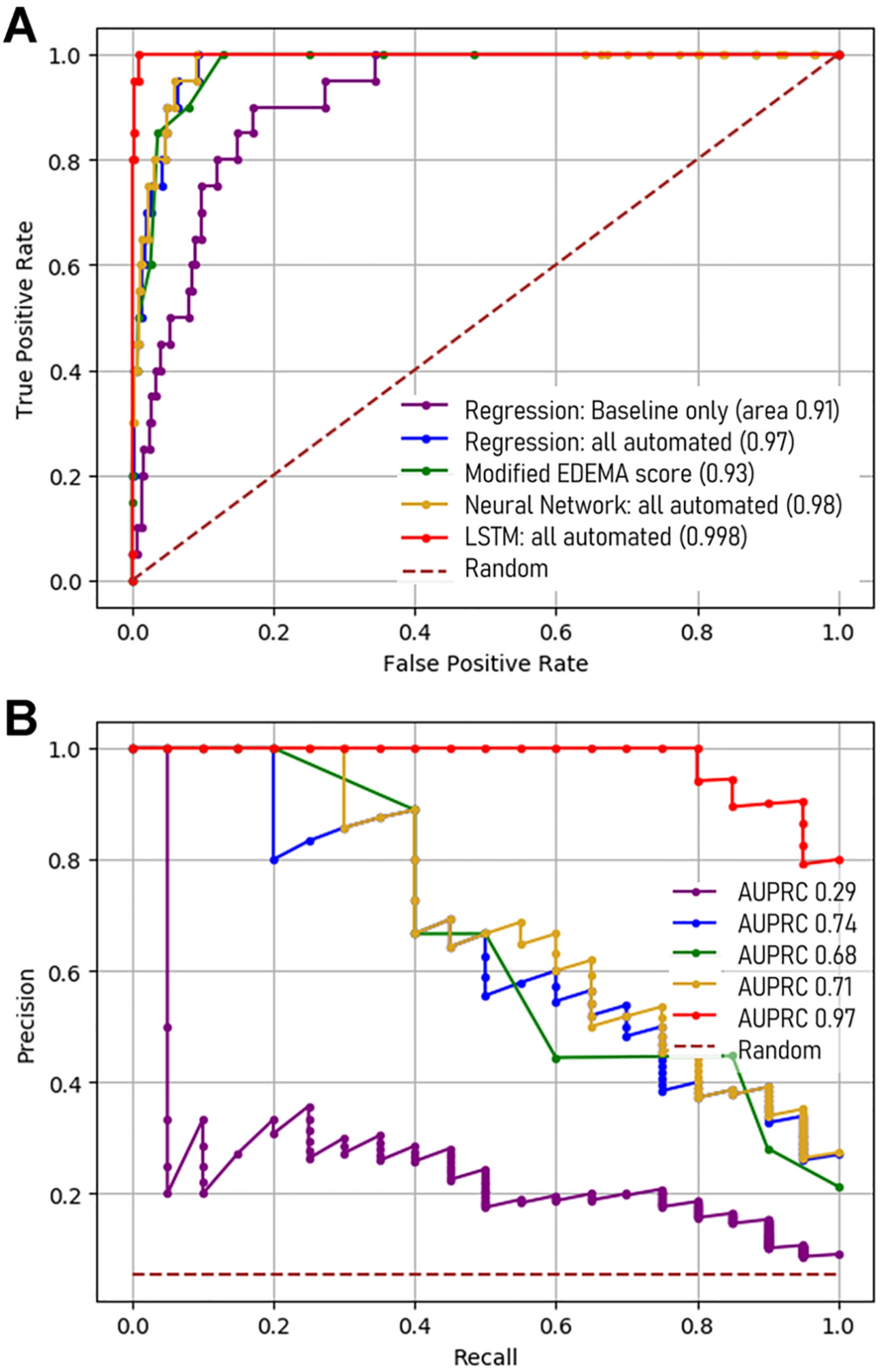
Performance curves for the regression and neural network models, as well as the modified EDEMA score, for predicting malignant edema over the range of thresholds. (A) Area under receiver operating characteristic (AUROC) and (B) precision-recall (AUPRC) curves.

A fully connected neural network was not able to significantly improve prediction when incorporating all automated features. Recall was identical to the regression model at 95% and precision was marginally better (33 vs. 32%). In contrast, when an LSTM model was trained on the same data, recall improved to 100% (i.e. all cases were correctly identified) and precision improved to 87%, resulting in an AUPRC of 0.97 (FIGURE 4, p=0.0001 for superiority, compared with the regression and fully connected neural network models using the same data). Incorporating midline shift and infarct volume did not improve precision or performance of the LSTM prediction model. Performance of these models was very similar in the subgroup of stroke patients with NIHSS ≥ 8 (full results shown for this subgroup and additional metrics provided in Supplemental Table 1). The LSTM model with all automated features still had superior prediction to that provided by the regression model (precision of 87% vs. 33%, AUPRC 0.97 vs. 0.74, p=0.001) in this high-risk subgroup.

**Figure 5:**
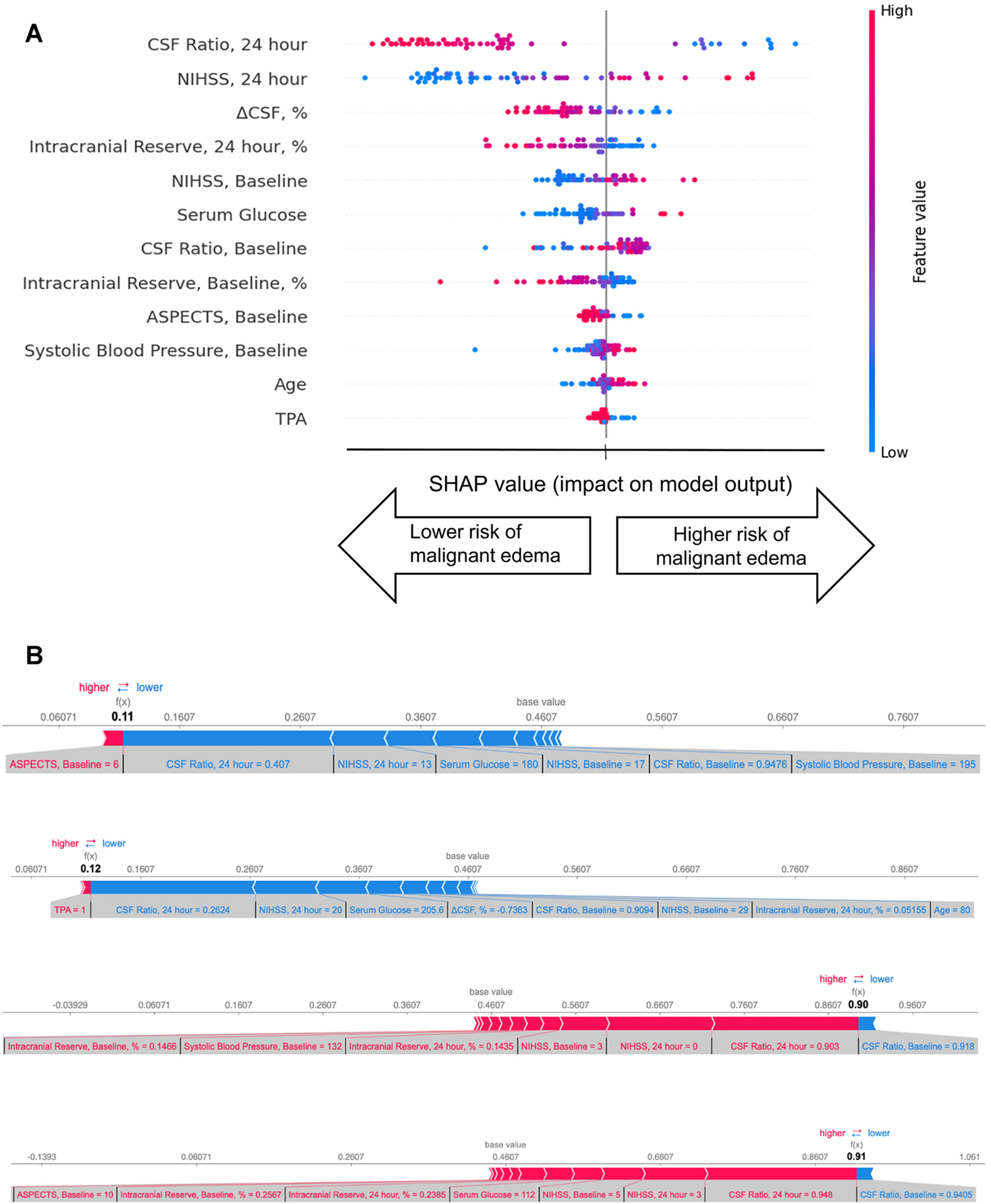
SHAP plots for the LSTM prediction model. (A) Summary plot: features are ranked in descending order of importance. For each feature, the horizontal location of each dot indicates whether the effect of that particular value is associated with higher or lower prediction of malignant edema. Each value is color-coded from red (high values) to blue (low values) within each feature. (B) Individual SHAP force plots for two cases with malignant edema and two controls, demonstrating how features influence final model prediction. Higher values (to the right) indicate higher probability of not developing edema. The base prediction (0.46) is the starting point, when no features are known. Blue arrows indicate features contributing to higher risk of malignant edema (i.e. shift probability to the left). The relatively importance is designated by the size of the arrow and the value of each feature is shown below each arrow. In the top two cases, low CSF ratio and high NIHSS at 24-hours shift probability left, toward prediction of malignant edema. The final probability is shown at the intersection of blue and red (under f(x)): in these two cases it is 0.11 and 0.12, translating into a predicted probability of 0.88 and 0.89 that these two patients will develop malignant edema. The red arrows in the lower two cases indicate features that shift probability to the right (i.e. not having malignant edema): these include higher intracranial reserve, higher CSF ratio and lower NIHSS at 24-hours. Predicted probabilities were 0.90 and 0.91, and both were correctly identified as not developing malignant edema.

The features in the LSTM model with the greatest contribution to predicting malignant edema are shown in the SHAP summary plot (FIGURE 5A). This demonstrates that the hemispheric CSF ratio and NIHSS at 24-hours had the greatest contributions to shifting the predicted probability of malignant edema. Despite its correlation with CSF ratio, ΔCSF provided additional predictive value. Lower intracranial reserve and higher glucose values also increased the risk while other variables, like age and ASPECT score, did not contribute much to prediction. The influence of these features on individual patients could be extracted to provide personalized and explainable predictions for malignant edema, as outlined in four patient examples (FIGURE 5B).

## DISCUSSION:

The evolution of cerebral edema depends on many factors beyond stroke severity and lesion size. These may include depth of ischemia related to lack of collaterals and biologic factors such as hyperglycemia [31, 32]. Genetic factors, most as yet undetermined, likely also influence edema formation [33]. Predicting which stroke patients will develop malignant edema severe enough to precipitate deterioration and necessitate hemicraniectomy is a critical challenge [3]. The American Heart Association recommends non-contrast CT as the first-line diagnostic test for monitoring edema, but acknowledges that accurate means of triage to surgery do not currently exist [34]. Radiographic signs of large stroke-related hypodensity on CT or large diffusion-weighted imaging lesion volume have reasonable accuracy but, depending on the threshold, low sensitivity and/or specificity [4, 6]. Furthermore, biomarkers that focus on the size of the visible lesion require manual measurement and do not separately quantify the edema component that primarily contributes to deterioration.

One of the earliest radiographic correlates of evolving edema is the effacement of CSF-filled sulci in the hemisphere of infarction. We have demonstrated that displacement of CSF provides a relevant quantitative biomarker of edema that can be extracted automatically from routine CT scans [15]. We have also demonstrated that incorporating quantitative CT-derived biomarkers improves prediction of malignant edema [16]. However, a regression model using ΔCSF provided only 90% sensitivity while precision remained even lower, meaning several stroke patients would be misclassified and potentially could be incorrectly sent for surgery.

The current proof-of-principle study demonstrates the potential of two significant advances to that prior work: first, we automatically extracted all quantitative imaging features utilized for prediction in a single workflow (as shown in Figure 1). Our primary models were constructed without traditional imaging markers that must be manually measured: that is, they did not require midline shift or infarct volume, instead focusing on CT-based CSF volumetrics [35]. The deep learning-based segmentation allowed us to extract these imaging parameters from paired routine CT scans of almost six hundred stroke patients enrolled in a prospective study at three international stroke centers. This high throughput data acquisition then facilitated the second innovation, employing a recurrent neural network to assist with prediction of a relatively rare but critical event. Only twenty patients in our cohort, which included hemispheric strokes of varying severities, required DHC or died from malignant edema. No individual variables, or even linear combination of variables from 24-hour imaging, could fully delineate all those with malignant outcomes from controls with similar data (as shown in the overlap in FIGURE 2).

Combining clinical and all automated imaging features into a regression model improved performance beyond that provided by baseline features alone. Although this model could identify 95% of cases with AUROC of 0.98, it still exhibited low precision. This illustrates how accuracy and even AUROC can be artificially inflated by high number of negative controls, and why precision and recall are more appropriate metrics for such imbalanced datasets [28]. The precision and AUPRC were not improved when the same data was incorporated into a fully connected neural network but were significantly higher using the LSTM model (FIGURE 4); notably, the AUROC had achieved a ceiling and was only marginally improved despite much better performance with the LSTM (FIGURE 3). In fact, this recurrent neural network was able to detect all cases of malignant edema with many fewer false positives, even when using only the automated, CSF-based data; addition of traditional manually-obtained imaging markers such as midline shift did not further improve prediction. It also maintained high precision when tested in the subset with more severe strokes. This LSTM model is able to analyze longitudinal data in ways that are more complex, incorporating both non-linear interactions and the temporal dimension of the data in making predictions. This type of deep learning approach has been demonstrated to have superior performance for time-series data and predicted post-stroke pneumonia better than other machine learning models [27]. It has also been the most promising approach for developing complex prediction models in the intensive care environment, where data is often collected on critically ill patients in a longitudinal manner [29, 36].

This is the first study to demonstrate such high precision for edema prediction, approaching the level of certainty that would be required to make confident treatment decisions on these patients by 24-hours, prior to deterioration or development of significant midline shift. In comparison, validated edema nomograms were able to perform similarly to our best regression model (i.e. high sensitivity but low predictive power) [12, 30]. Application of such nomograms could, at best, triage high-risk patients to closer monitoring but would not allow confident surgical decision-making. Rather than relying on close neurologic monitoring and waiting for signs of impending herniation to consider surgery, we suggest that 24-hour CT be performed in any patients with moderate risk: for example, those whose NIHSS remains elevated. The quantitative data extracted from that CT (along with data from baseline CT and clinical variables) could be fed into the deep learning algorithm and a probability of malignant edema would be provided to the clinician (as shown in FIGURE 1 for a real case). This framework could be employed for clinician decision-support to improve the triage of stroke patients, avoiding risk of secondary damage when surgery is performed after midline shift and herniation has already developed.

There remain several limitations to our approach. We applied machine learning to develop and test a complex prediction model. There is concern that such multi-layered models may overfit the training data and suffer from limited generalizability. This is especially true given the relatively low number of patients with the outcome-of-interest in our cohort (i.e. imbalanced dataset). We acknowledge that application of our approach does require validation in external cohorts, ideally with prospective imaging of all at-risk patients, rather than the selected subgroup we analyzed. However, we do not believe that the impressive performance of our model is due to overfitting, as we employed several well-accepted methods to minimize this risk such as regularization, drop-out, and early stopping. The results that we present were obtained using stratified cross-validation, an approach that ensures representative numbers of cases are in each fold and tests each model only on new and unseen data that was not used to train or tune the model. The relatively low incidence of malignant edema mirrors that in real-world stroke populations [37], which minimizes the risk of dataset shift, a phenomenon that imperils the application of machine learning models to real-world clinical practice [38]. In addition, our dataset was comprised of stroke patients from three stroke centers in different nations with different practices, suggesting that the model does not simply reflect a single institution’s practice in managing edema or selecting patients for surgery. Nonetheless, the cohort was limited to those enrolled and who had imaging performed, which could limit generalizability. We did not perform external validation. Further external training and testing in large prospective cohorts is required before broad adoption.

A second critique of models derived from neural networks is that they are typically ‘black box’ and lack transparency in which features are driving the predictions being provided. This can limit trust in their predictions and hinder adoption of seemingly opaque algorithms. We utilized a relatively recently approach to provide greater interpretability. SHAP values apply insights derived from game theory to provide a measure of how much each feature shifts the prediction, in coalition with all other factors [39]. While not the same as individual weights in a traditional regression model, they provide a sense of the relative importance of features in the context of their complex non-linear interactions. They provide both global interpretability, i.e. how each predictor collectively contributes to outcome prediction across the entire dataset, as well as local interpretability, i.e. in each patient, a transparent read-out of how each variable was utilized to make individual predictions. In that way, we were able to demonstrate that a combination of 24-hour NIHSS and the hemispheric CSF volume ratio (i.e. how much CSF remained in the hemisphere of the stroke divided by the volume in the contralateral side, especially if below 0.60) contributes most to prediction of malignant edema.

Several means of improving this promising approach remain. Although we utilized a large dataset of almost six hundred stroke patients from three sites, the number of outcomes was still relatively small. Maximizing the strength of neural networks requires even larger datasets and ideally more longitudinal measurements (e.g. hemodynamic and neurologic status). Incorporation of multimodal imaging, including perfusion parameters, could further enhance prediction of edema. MRI assessment of lesion volume, though not as accessible as CT, could improve prediction of edema at earlier time points. Using more complex network architectures, we could even feed the entire raw imaging data into a multimodal model, rather than extracting quantitative features first; this would likely require much larger datasets and is the subject of ongoing investigation [40]. At this point, our imaging algorithm is still optimized for research purposes and not for the bedside. Further refinements are required to create an end-to-end platform that can seamlessly analyze imaging data and provide a rapid, confident prediction of whether a patient will develop edema.

## Summary/Conclusions

We demonstrated that a LSTM neural network can incorporate volumetric data that is extracted automatically from baseline and 24-hour CT imaging and provide highly precise predictions of which patients will require surgery or die from malignant edema. This deep learning framework substantially outperformed a fully connected neural network, traditional regression models and the validated EDEMA nomogram for predicting cases of malignant edema. Our approach is also transparent, providing outputs from the neural network that explain how each individual prediction was made. Stroke severity and asymmetry of hemispheric CSF volumes on routine CT at 24-hours contributed the most to prediction of malignant edema. With further validation in prospective cohorts, this automated approach could assist in the accurate selection of which patients will require surgery, prior to deterioration.

## Supplementary Material

1737973_Sup_tab.

## Figures and Tables

**Table 1:** Comparison of baseline and follow-up features in cases with malignant edema and controls

Variable	Malignant Edema (n=20)	No Malignant Edema (n=578)	P value
**Age, years, mean (SD)**	65.3 (15)	68.9 (13)	0.32
**Sex, female**	13 (65%)	264 (46%)	0.23
**Race, non-white**	5 (25%)	51 (9%)	0.04
**NIHSS score, baseline, median (IQR)**	18.5 (15.5–21)	9 (4–15)	< 0.0001
**Serum glucose, mg/dl, mean (SD)**	202 (109)	135 (51)	0.01
**Systolic blood pressure, mm Hg, mean (SD)**	168 (37)	160 (28)	0.47
**Received tPA**	15 (75%)	454 (79%)	0.92
**Endovascular intervention**	4 (20%)	98 (17%)	0.97
**Time from stroke onset to baseline CT, hours, median (IQR)**	1.4 (1.1–3.8)	1.7 (1.1–3.0)	0.97
**ASPECTS on baseline CT, median (IQR)**	8 (6–10)	10 (9–10)	0.0002
**Intracranial reserve, mean (SD)**	10.4% (4)	14.0% (5)	0.002
**Hemispheric CSF ratio, baseline, median (IQR)**	0.90 (0.85–0.93)	0.93 (0.87–0.97)	0.05
**Time from stroke onset to FU CT, hours, median (IQR)**	22.4 (16.7–37.1)	25.3 (19.8–27.9)	0.78
**NIHSS at 24-hours, median (IQR)**	20.5 (19–26)	5 (2–11)	< 0.0001
**Change in NIHSS, mean (SD)**	+3 (7)	−3 (7)	0.0009
**Midline shift at 24-hours, mm, median (IQR)**	4.7 (3.3–9.7)	0 (0–0)	< 0.0001
**Infarct hypodensity volume at 24-hours, ml, median (IQR)**	253 (181–322)	0 (0–22)	< 0.0001
**Hemispheric CSF Ratio at 24- hours, median (IQR)**	0.26 (0.11–0.38)	0.90 (0.77–0.95)	< 0.0001
**Reduction in CSF volume %, mean (SD)**	52% (18)	15% (16)	< 0.0001
**EDEMA score, median (IQR)**	7 (4–10)	1 (1–2)	< 0.0001
**Modified EDEMA score, median (IQR)**	9 (7–11)	1 (1–4)	< 0.0001

Abbreviations: ASPECTS, Alberta Stroke Program Early CT Score; EDEMA, Enhanced Detection of EDEma in Malignant Anterior circulation stroke; NIHSS, National Institutes of Health Stroke Scale
