# Supplementary material for: Accelerating Prediction of Malignant Cerebral Edema after Ischemic Stroke with Automated Image Analysis and Explainable Neural Networks
Source: Neurocrit Care. Author manuscript; Available in PMC 2023 Apr 1. (PMC8858326; doi:10.1007/s12028-021-01325-x)
Supplement: 1737973_Sup_tab. [file NIHMS1737973-supplement-1737973_Sup_tab_.docx]

**SUPPLEMENTAL MATERIAL**

**Supplemental Methods: Details of model development and testing**

Values for NIHSS, glucose, blood pressure, and CSF ratio were missing in nine (2%), 13 (2%), 37 (6%), and 5 (1%) of cases. These were imputed using the five patients with closest data points, using the K-Nearest Neighbor algorithm. All features were then standardized to improve stability of training and model robustness. The full dataset was partitioned into ten folds using stratified and nested cross validation to train and then test each model. In each of ten rounds, the 9-folds dataset was partitioned into training and inner-validation sets with cases of malignant edema oversampled to balance cases and controls within the training portion. Training size was further increased using data augmentation: i.e. adding uniform noise of zero mean and 0.08% variance. After training each model, performance was independently evaluated on the original, unaltered patient data not seen by algorithm during training or tuning. This process was repeated ten times such that each fold was used once for testing. All models were implemented within the Keras machine-learning platform within Python. In all models, parameters were optimized by minimizing the cross entropy loss function. Hyperparameters were optimized with grid search and based on performance on inner validation sets. Logistic regression models were built using a single layer network with linear inputs and softmax activation. The fully connected neural network had one hidden layer with 32 neurons and L1 (0.05) regularization was used in its hidden layer and output layer. The number of hidden layers and units for each layer of the LSTM model were optimized with inner cross-validation while dropout, recurrent dropout (both set at 0.2) and cross-validation were used to prevent over-fitting [1]. A ReLu activation function was used for all layers, except for the output layer. Batch size was 32 and learning rate was 0.0005. The final LSTM model had two hidden layers, the first with 32 LSTM cells and the second with 8 dense units, as shown in Supplemental Figure 1.

A confusion matrix was constructed for each model representing predictions and actual outcomes, showing true positives (TP), true negatives (TN), false positives (FP), and false negatives (FN). Recall is sensitivity to detect cases of malignant edema (TP/TP+FN). Precision is the positive predictive value (TP/TP+FP). Accuracy is the ratio of correct predictions to total observations (TP+TN/TP+FP+FN+TN); in imbalanced datasets with many negative controls it overestimates predictive performance for cases. The F1 score is the weighted average of precision and recall: 2*(Recall * Precision)/(Recall + Precision). It takes both false positives and false negatives into account and is generally preferred to accuracy in imbalanced datasets. We also provide the Brier score, the mean squared error between predicted probabilities and expected values, with a lower value (closer to 0) meaning a prediction model with better discrimination.

**Supplemental Table 1**: **Comparison of performance between models for prediction of malignant cerebral edema in all strokes and in the subgroup with NIHSS equal to or greater than eight**

| **Model** | **Recall (Sensitivity)** | **Precision (Predictive Value)** | **F1 Score** | **Specificity** | **Accuracy** | **AUROC** | **Std Dev (AUROC)** | **AUPRC** | **Std Dev (AUPRC)** | **Brier Score** | **Std Dev (Brier)** |
| --- | --- | --- | --- | --- | --- | --- | --- | --- | --- | --- | --- |
| **Regression: baseline only** | 0.85 | 0.15 | 0.26 | 0.83 | 0.83 | 0.91 | 0.07 | 0.29 | 0.23 | 0.115 | 0.022 |
| **Subgroup: NIHSS ≥ 8** | 0.85 | 0.09 | 0.16 | 0.7 | 0.71 | 0.85 | 0.12 | 0.29 | 0.23 | 0.189 | 0.026 |
| **Regression: baseline + ΔCSF** | 0.9 | 0.24 | 0.38 | 0.9 | 0.9 | 0.97 | 0.03 | 0.66 | 0.17 | 0.074 | 0.0212 |
| **Subgroup: NIHSS ≥ 8** | 0.9 | 0.26 | 0.40 | 0.83 | 0.84 | 0.95 | 0.04 | 0.67 | 0.15 | 0.117 | 0.029 |
| **Regression: automated^†^** | 0.95 | 0.32 | 0.48 | 0.93 | 0.93 | 0.98 | 0.02 | 0.74 | 0.29 | 0.05 | 0.012 |
| **Subgroup: NIHSS ≥ 8** | 0.95 | 0.33 | 0.49 | 0.87 | 0.88 | 0.97 | 0.03 | 0.74 | 0.29 | 0.09 | 0.025 |
| **Regression: all features** | 0.95 | 0.34 | 0.50 | 0.94 | 0.94 | 0.98 | 0.02 | 0.63 | 0.32 | 0.086 | 0.026 |
| **Subgroup: NIHSS ≥ 8** | 0.95 | 0.35 | 0.51 | 0.89 | 0.89 | 0.97 | 0.03 | 0.64 | 0.31 | 0.05 | 0.013 |
| **EDEMA score** | 0.9 | 0.28 | 0.43 | 0.92 | 0.92 | 0.91 | 0.12 | 0.66 | 0.24 | 0.03 | 0.007 |
| **Subgroup: NIHSS ≥ 8** | 0.9 | 0.34 | 0.49 | 0.88 | 0.88 | 0.89 | 0.12 | 0.68 | 0.23 | 0.05 | 0.013 |
| **Modified EDEMA score** | 1 | 0.22 | 0.36 | 0.87 | 0.87 | 0.93 | 0.014 | 0.68 | 0.22 | 0.04 | 0.006 |
| **Subgroup: NIHSS ≥ 8** | 1 | 0.22 | 0.36 | 0.76 | 0.77 | 0.88 | 0.031 | 0.68 | 0.22 | 0.06 | 0.01 |
| **Neural Network: automated^†^** | 0.95 | 0.33 | 0.49 | 0.93 | 0.93 | 0.98 | 0.18 | 0.71 | 0.28 | 0.05 | 0.013 |
| **Subgroup: NIHSS ≥ 8** | 0.95 | 0.34 | 0.50 | 0.88 | 0.93 | 0.97 | 0.03 | 0.71 | 0.28 | 0.088 | 0.024 |
| **LSTM: automated features^†^** | 1 | 0.87 | 0.93 | 0.99 | 0.99 | 0.998 | 0.005 | 0.97 | 0.06 | 0.008 | 0.014 |
| **Subgroup: NIHSS ≥ 8** | 1 | 0.87 | 0.93 | 0.99 | 0.99 | 0.999 | 0 | 0.97 | 0.06 | 0.013 | 0.025 |
| **LSTM: all features** | 0.95 | 0.86 | 0.90 | 0.99 | 0.99 | 0.998 | 0.005 | 0.94 | 0.11 | 0.007 | 0.012 |
| **Subgroup: NIHSS ≥ 8** | 0.95 | 0.86 | 0.90 | 0.99 | 0.98 | 0.997 | 0.02 | 0.94 | 0.11 | 0.01 | 0.022 |

^†^ all automated features without infarct volume or midline shift; cutoff for EDEMA score was 4 and for modified EDEMA score was 5.

NB: Models presented in main manuscript figures are labeled using same color scheme (in first column).

Metrics’ columns are color coded using a white-red color scale to highlight cells with highest (dark red) and lowest (white) values. Brier scale is coded using a blue-white-red scale where blue values are highest (worst performance) and red are lowest values (best performance)

Abbreviations: AUROC, area under receiver-operating-characteristic; AUPRC, area under precision-recall curve; LSTM, long short-term memory; Std Dev, standard deviation.

**
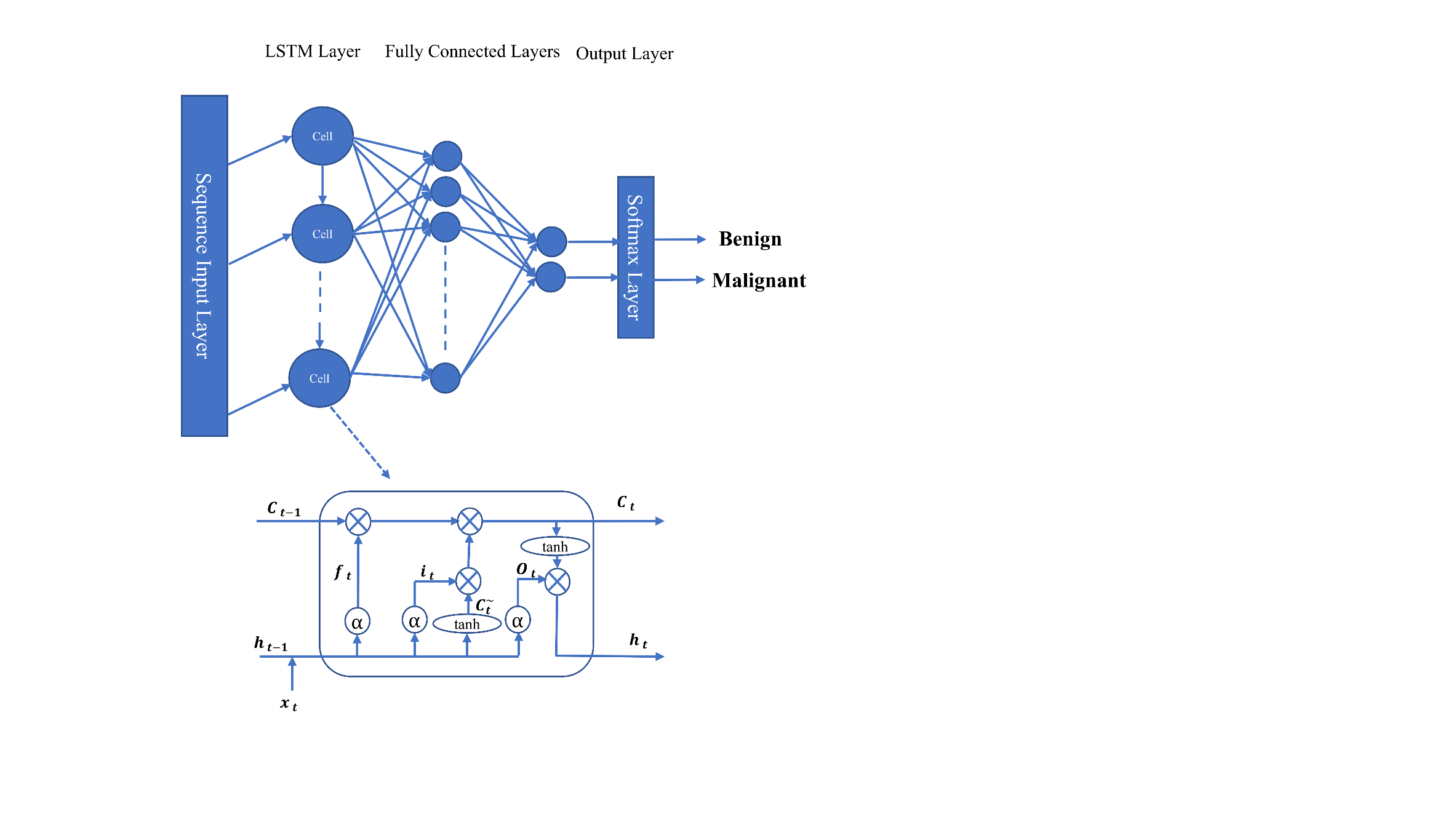
Supplemental Figure 1: Architecture of the long short-term memory (LSTM) neural network used for prediction**

The model utilized had two hidden layers, the first with 32 LSTM cells and the second with 8 dense units. Baseline and follow-up features are fed into LSTM cells which learn meta features from time series data. These are then fed into the dense layer to learn nonlinearity between features before being fed ino the last softmax layer for providing output probabilities of each class (malignant vs. non-malignant, benign edema).

**Supplemental Figure 2: Flow of study participants into imaging prediction study**


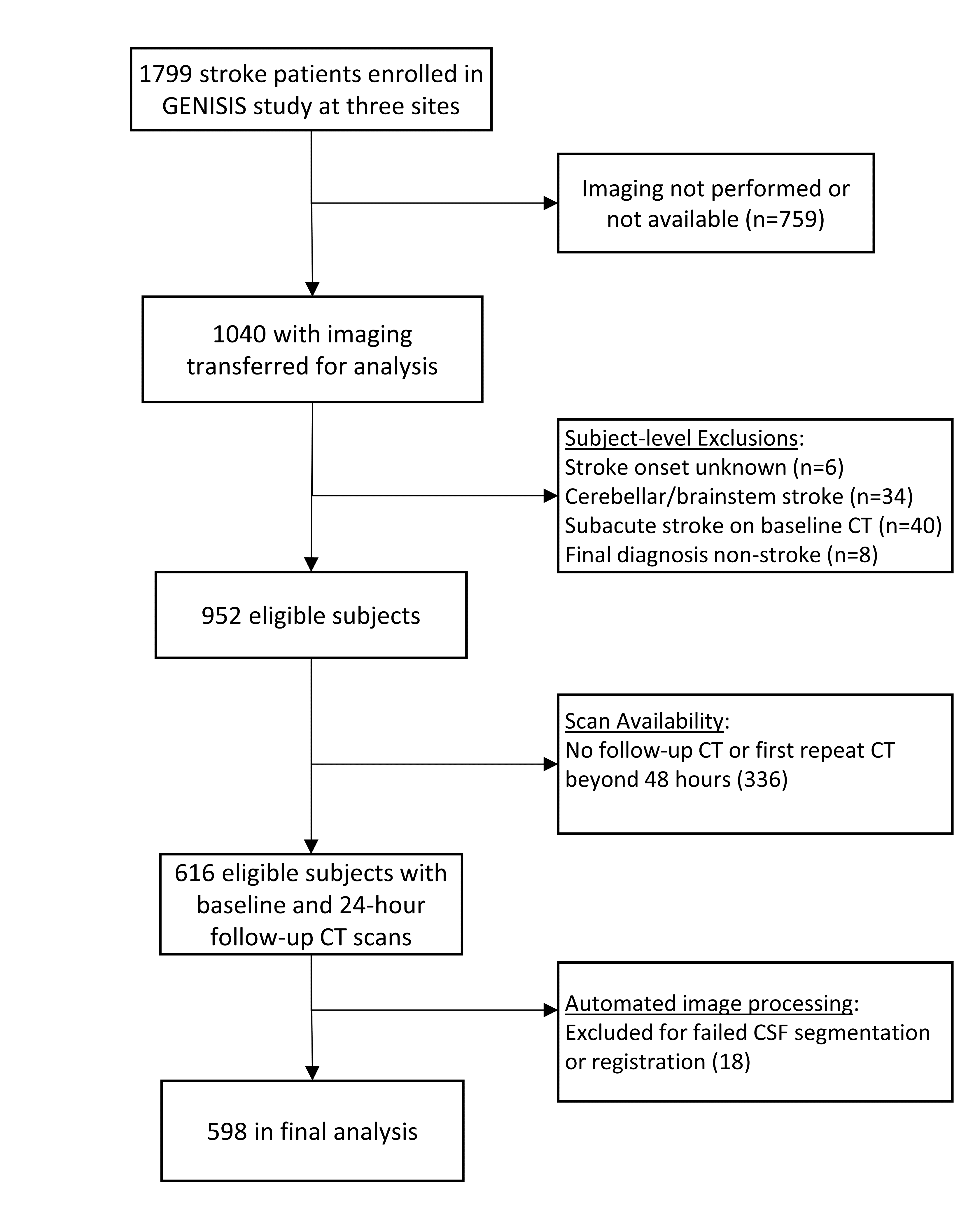


**Supplemental References**

1. Srivastava, N., Hinton, G., Krizhevsky, A., Sutskever, I.Salakhutdinov, R. Dropout: A Simple Way to Prevent Neural Networks from Overfitting*.* Journal of Machine Learning Research 2014;15:1929-58.
